# A multi-vendor, multi-center study on reproducibility and comparability of fast strain-encoded cardiovascular magnetic resonance imaging

**DOI:** 10.1007/s10554-020-01775-y

**Published:** 2020-02-13

**Authors:** Jennifer Erley, Victoria Zieschang, Tomas Lapinskas, Aylin Demir, Stephanie Wiesemann, Markus Haass, Nael F Osman, Orlando P Simonetti, Yingmin Liu, Amit R Patel, Victor Mor-Avi, Orhan Unal, Kevin M Johnson, Burkert Pieske, Jochen Hansmann, Jeanette Schulz-Menger, Sebastian Kelle

**Affiliations:** 1 Department of Internal Medicine/Cardiology, German Heart Institute Berlin, Augustenburger Platz 1, 13353 Berlin, Germany; 2 Department of Cardiology, Medical Academy, Lithuanian University of Health Sciences, Kaunas, Lithuania; 3 Working Group Cardiovascular Magnetic Resonance, Experimental and Clinical Research Center, Max-Delbrueck Center for Molecular Medicine, Department of Cardiology and Nephrology, Charité Medical Faculty, HELIOS Klinikum Berlin Buch, Berlin, Germany; 4 Department of Internal Medicine/Cardiology/Angiology, Theresienkrankenhaus Und St. Hedwig-Klinik, Mannheim, Germany; 5 Department of Radiology and Radiological Science, School of Medicine, John Hopkins University, Baltimore, MD USA; 6 Myocardial Solutions, Inc, Morrisville, NC USA; 7 Departments of Internal Medicine and Radiology, The Ohio State University, Columbus, OH USA; 8 Dorothy M. Davis Heart and Lung Research Institute, Wexner Medical Center, The Ohio State University, Columbus, OH USA; 9 Department of Cardiology, University of Chicago Medicine, Chicago, IL USA; 10 Departments of Radiology and Medical Physics, University of Wisconsin-Madison, Madison, WI USA; 11 Department of Internal Medicine/Cardiology, Charité Campus Virchow Klinikum, Berlin, Germany; 12 DZHK (German Center for Cardiovascular Research), Partner Site Berlin, Berlin, Germany; 13 Department of Radiology, Theresienkrankenhaus Und St. Hedwig-Klinik, Mannheim, Germany

**Keywords:** Strain, fSENC, Agreement, Reproducibility, CMR, Cardiac, Magnetic resonance

## Abstract

Myocardial strain is a convenient parameter to quantify left ventricular (LV) function. Fast strain-encoding (fSENC) enables the acquisition of cardiovascular magnetic resonance images for strain-measurement within a few heartbeats during free-breathing. It is necessary to analyze inter-vendor agreement of techniques to determine strain, such as fSENC, in order to compare existing studies and plan multi-center studies. Therefore, the aim of this study was to investigate inter-vendor agreement and test-retest reproducibility of fSENC for three major MRI-vendors. fSENC-images were acquired three times in the same group of 15 healthy volunteers using 3 Tesla scanners from three different vendors: at the German Heart Institute Berlin, the Charité University Medicine Berlin-Campus Buch and the Theresien-Hospital Mannheim. Volunteers were scanned using the same imaging protocol composed of two fSENC-acquisitions, a 15-min break and another two fSENC-acquisitions. LV global longitudinal and circumferential strain (GLS, GCS) were analyzed by a trained observer (Myostrain 5.0, Myocardial Solutions) and for nine volunteers repeatedly by another observer. Inter-vendor agreement was determined using Bland-Altman analysis. Test-retest reproducibility and intra- and inter-observer reproducibility were analyzed using intraclass correlation coefficient (ICC) and coefficients of variation (CoV). Inter-vendor agreement between all three sites was good for GLS and GCS, with biases of 0.01–1.88%. Test-retest reproducibility of scans before and after the break was high, shown by ICC- and CoV values of 0.63–0.97 and 3–9% for GLS and 0.69–0.82 and 4–7% for GCS, respectively. Intra- and inter-observer reproducibility were excellent for both parameters (ICC of 0.77–0.99, CoV of 2–5%). This trial demonstrates good inter-vendor agreement and test–retest reproducibility of GLS and GCS measurements, acquired at three different scanners from three different vendors using fSENC. The results indicate that it is necessary to account for a possible bias (< 2%) when comparing strain measurements of different scanners. Technical differences between scanners, which impact inter-vendor agreement, should be further analyzed and minimized.

DRKS Registration Number: 00013253.

Universal Trial Number (UTN): U1111-1207-5874.

## Introduction

Myocardial strain has proven to be an important parameter for further investigation of myocardial performance in addition to conventionally used volumetric measures, such as ejection fraction (EF) [1–3]. Strain can be determined using echocardiography and cardiovascular magnetic resonance (CMR) imaging. A common technique to measure strain in echocardiography is using speckle tracking (STE). STE is routinely used, for example to identify systolic dysfunction in heart failure patients with preserved EF [4] or as a marker for cardiotoxicity in patients undergoing chemotherapy [5]. An important step towards standardization of STE in preparation for broad clinical use was the recent publication of a consensus document on how strain measurements should be performed [6]. However, strain is not only influenced by measurement methods, but also by image quality, intra- and inter-observer reproducibility, the image acquisition system [7] and the post-processing software used [8, 9]. As the impact of these various factors on strain results remains unclear, guidelines recommend STE to be performed using the same vendor’s acquisition system and software for individual patients [9].

As CMR emerged as the reference standard of cardiac morphology and function [10], various acquisition- and post-processing techniques to determine strain using CMR have been explored and validated [11]. Long acquisition times [12] and long breath-holds in patients with cardiac diseases, especially those who suffer from dyspnea, are some of the factors currently limiting use in clinical settings. Furthermore, no standardized approach to measure strain using CMR has been proposed yet, as was the case for STE. The lack of information on the influence of different magnetic resonance scanners and platforms on strain results is one challenge preventing standardization of CMR techniques. Nevertheless, this information is crucial since studies are conducted at different centers with varying scanners, at different field strengths and using different post-processing platforms. Without information on inter-vendor agreement, CMR-strain should only be determined using the same scanner and post-processing software for individual patients, as recommended for STE. Although this measure reduces possible bias on strain results, no comparison can be made between different studies and measurements performed at different centers, hampering the practicality of using strain routinely and the design of multi-center studies to further validate this method.

Strain-encoding (SENC), first described in 2001 by Osman et al. [13], is a novel imaging technique to measure strain. In comparison to myocardial tagging, SENC uses tag planes in which the sinusoidal phase is constant in parallel to the image plane [13]. Therefore, longitudinal strain is determined using short-axis- and circumferential strain using long-axis views; radial strain is not measurable by SENC. Fast-SENC (fSENC) is a “real-time” scan that acquires all necessary data for one slice within one single heartbeat [14]. Hence, it is insensitive to breathing motion, resulting in a fast magnetic resonance imaging (MRI) exam for the patient at free breathing. Studies have shown that fSENC is equal or even superior to tagging [15] and highly reproducible concerning inter-study, as well as intra- and inter-observer reproducibility [2].

The aim of this study was to examine the inter-vendor agreement and reproducibility of CMR-derived strain, obtained with fSENC in the same group of volunteers at three different sites with individual MRI-platforms and sequences. In particular, our aims were to

1. investigate inter-vendor agreement of fSENC at 3 T using scanners from three major MRI vendors,
2. determine test-retest reproducibility of repeated scans at each scanner and
3. determine intra- and inter-observer reproducibility of the strain measurements.

## Methods

### Study population and design

Fifteen healthy volunteers with no history of cardiovascular diseases or contraindications against MRI [16] were prospectively identified and recruited for the study after obtaining a written informed consent. The study was approved by the Ethics Committee of the Charité-University-Medicine in Berlin and complied with the Declaration of Helsinki. It was registered at the German Register for Clinical Studies (DRKS) (registration number: 00013253) and the World Health Organization (WHO) (universal trial number (UTN): U1111-1207-5874).

### Cardiovascular magnetic resonance imaging

CMR images of all fifteen volunteers were acquired repeatedly at 3 T on three different scanners (names in alphabetical order and not according to site number: Ingenia, Philips, Best, The Netherlands; MAGNETOM Verio, Siemens Healthcare GmbH, Erlangen, Germany; SIGNA Architect, GE Healthcare, Milwaukee, WI, USA). CMR examinations took place within five months at: the German Heart Institute Berlin (site I), the Theresien-Hospital Mannheim (site II) and the Max-Delbrück Center for Molecular Medicine (MDC) in collaboration with Charité University Medicine Berlin-Campus Buch (site III), each equipped with one of the above listed scanners.

### Fast strain-encoding (fSENC)

**Fig. 1:**
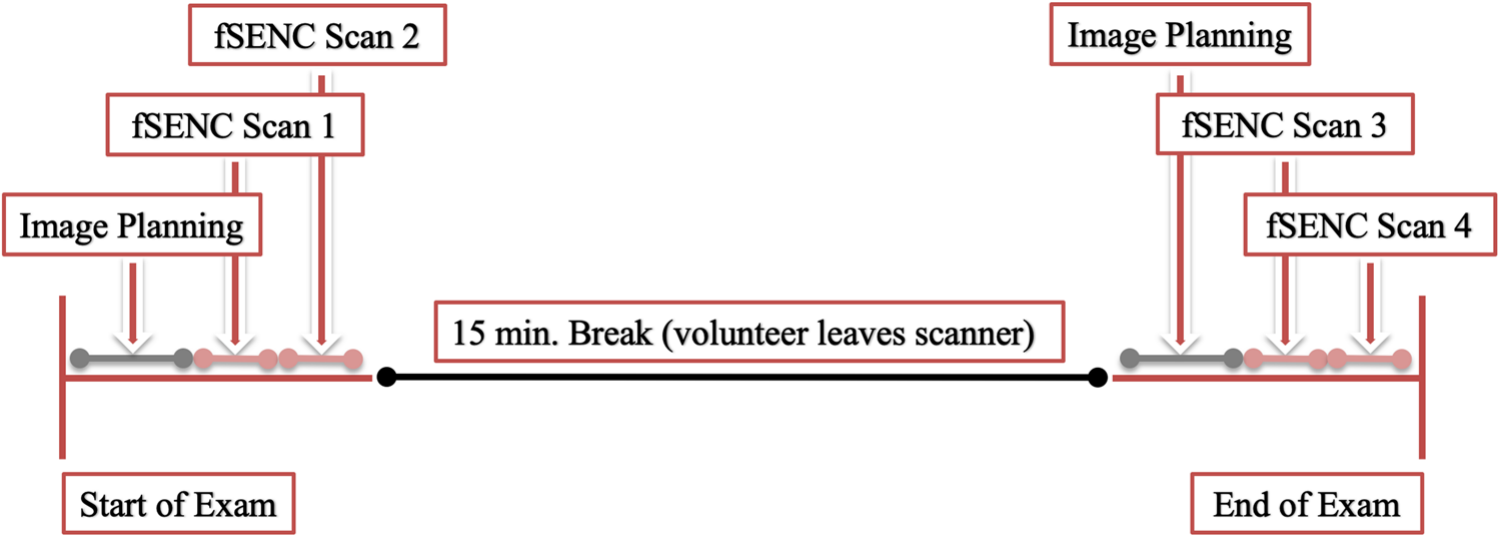
Schematic outline showing the scan organization with a total of four fSENC scans per volunteer at every site

The techniques applied to the pulse sequence (localized/reduced field-of-view fSENC, interleaved tuning, spiral imaging, ramped flip angle, etc.) to achieve image acquisition in a single heartbeat have been described previously [2, 14, 15]. Prior to in-vivo imaging, studies were performed in vitro with scanning platforms of the three different vendors using phantoms of very similar proportions, made of homogeneous MR-visible silicone gel with known mechanic properties [17]. Periodic non-flat compression and expansion was applied using an MR-compatible air cylinder as described by Osman et al. [17, 18]. Subsequently, scanning of the fifteen volunteers was performed at all three sites. All volunteers were scanned using the same imaging protocol, schematically depicted in Fig. 1. Each volunteer received four fSENC scans per site, adding up to 60 scans. The first two scans were performed consecutively using the same scanning parameters. Afterwards the volunteers left the scanner room for fifteen minutes, followed by two more fSENC acquisitions with the same parameters. Images were acquired in three long-axis views (2-chamber (2-ch), 3-chamber (3-ch), 4-chamber (4-ch)) to calculate left ventricular (LV) global circumferential strain (GCS) and in three short-axis views (SAX- basal, mid-ventricular (mid), apical) to calculate LV global longitudinal strain (GLS). Scanning was performed by the local team of one or two technicians at each site after being trained by the same representatives of the software provider on performing the fSENC acquisitions and completing a written test. Scanning parameters were allowed to be adjusted according to the different scanners, if needed. Heart rate (bpm) and blood pressure (mmHg) were monitored before, during and after the exam. Variables that might influence strain measurements, such as height, weight and smoking behavior, were determined before the scans at every site.

### Technical parameters

#### Site I

At site I, images were acquired using a multi-element receive coil array, consisting of an anterior part on the patient’s chest and a posterior part embedded in the patient table. A flexible number of up to 32 elements was employed, where the selection of coil elements was performed automatically by the MR software. Image acquisition was triggered on the R-wave using a 4-lead vector ECG. fSENC imaging parameters at site I were: field-of-view = 256 × 256 mm^2^, slice thickness = 10 mm, voxel size = 4 × 4 × 10 mm^3^, reconstructed images at 1 × 1 × 10 mm^3^ using zero-filled interpolation (in-plane ZIP 1024), spiral readout (3 interleaves) with acquisition time (TA) = 10 ms, flip angle = 30°, effective echo time (TE) = 0.7 ms, repetition time (TR) = 12 ms, temporal resolution = 36 ms, typical number of acquired heart phases = 22, spectrally selective fat suppression (SPIR), total acquisition time per slice < 1 s (1 heartbeat), total acquisition time per scan = 6 heartbeats.

#### Site II

At site II, a user-developed sequence was employed. Images were acquired using a multi-element receive coil array, as described for site I. fSENC spiral images were triggered on the R-wave using a 4-lead vector ECG. Field-of-view = 256 × 256 mm^2^, slice thickness = 7-8 mm, voxel size = 4 × 4 × 7 mm^3^, reconstructed images at 1 × 1 × 7 mm^3^, single-shot spiral readout (4 interleaves) with acquisition time (TA) = 7.5 ms, flip angle = 20°, effective echo time (TE) = 5.0 ms, repetition time (TR) = 9.1 ms, temporal resolution = 36.4 ms, typical number of acquired heart phases = 18, spectrally selective fat suppression (SPIR), total acquisition time per slice < 1 s (or one heartbeat), total acquisition time per scan = 6 heartbeats.

#### Site III

In comparison to the spiral pulse sequence at sites I and II, fSENC at site III is an Echo Planar Imaging (EPI) user-developed pulse sequence [19]. Volunteers were scanned using a 32-channel body coil and image acquisition was triggered on the R-wave using a 4-lead vector ECG. Epi-factor = 9, field-of-view = 450 × 170 mm^2^, slice thickness = 12 mm, voxel size = 4.7 × 4.7 × 12 mm^3^, reconstructed resolution at 4.7 × 4.7 × 12 mm^3^, flip angle = 12°, effective echo time (TE) = 1.18 ms, repetition time (TR) = 8.9 ms, temporal resolution = 35.6 ms, centric EPI recording, typical number of acquired heart phases = 22, spectrally selective fat suppression (SPIR). The acquisition happened in a single heartbeat, as for sites I and II. A separate heartbeat was used for EPI phase correction. The total acquisition time per slice was about 2 s (or two heartbeats) and per scan about 12 heartbeats.

### Image analysis

**Fig. 2:**
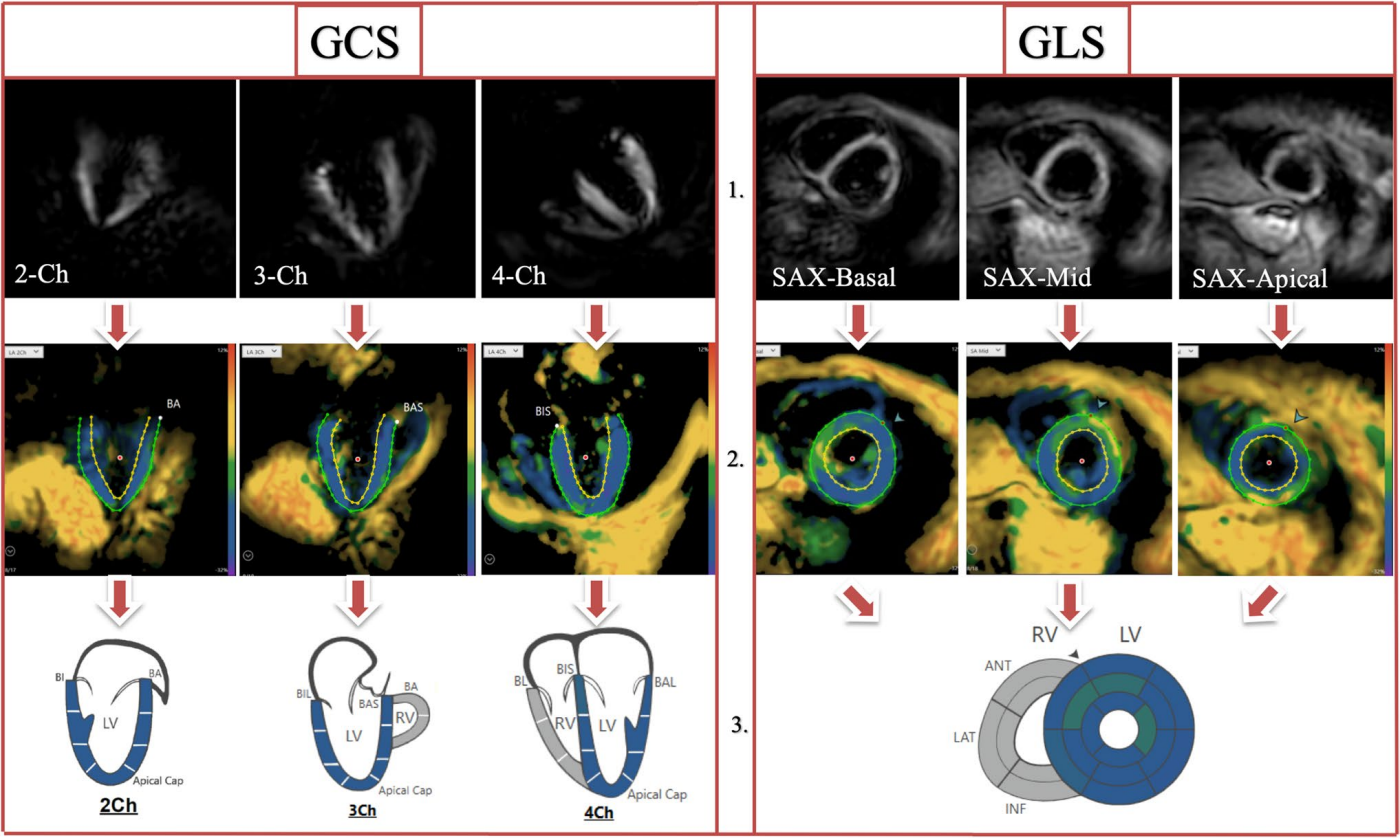
fSENC- and corresponding color-coded images after post-processing at end-systole (blue representing strain in the normal range during contraction), as well as the myocardial segmentation as illustrated by the software. Legend: 1. = Images as shown on the scanner, 2. = Color-coded images on the software after post-processing, displaying manually drawn epi-and endocardial contours at end-systole, 3. = Results of the strain analysis, represented by a color-coded map of the heart

**Fig. 3:**
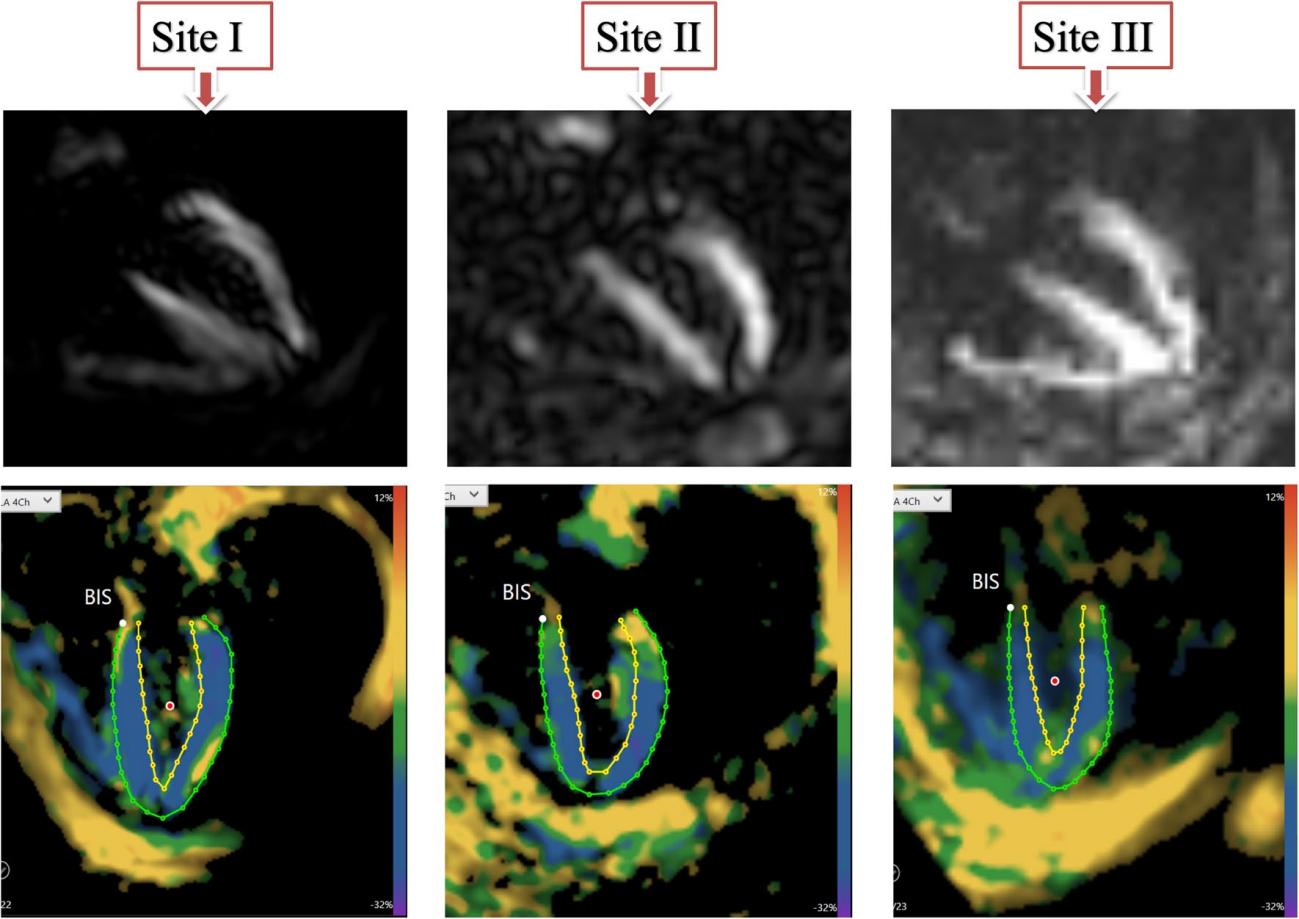
4-chamber view images of the same volunteer scanned at the three different sites, as shown on the scanner and after post-processing

All images were analyzed by one observer (JE) using dedicated software (Myostrain 5.0, Myocardial Solutions, Inc., Morrisville, North Carolina, USA), after being trained by a representative of the software company and completing a written test, as previously described [20]. Figure 2 demonstrates the process of image analysis, starting with the acquisition of the image on the scanner (1.), proceeding onto the color-coded image on the software, displaying the manually drawn endo- and epicardial contours at end-systole (2.) and onto the result of the strain analysis, represented by a color-coded map of the heart (3.). GCS was quantified in the three long-axis images by drawing epi- and endocardial contours manually at end-systole (as seen in Fig. 2), identified by the size of the heart and the color-coding of the images signaling contraction (blue). Papillary muscles and trabeculae were excluded from the endocardial contour. GLS was quantified using the short-axis images, again by drawing epi- and endocardial contours at end-systole (Fig. 2). The LV was automatically divided into 16 segments in the short-axis views and 21 segments in the long-axis views (according to the AHA model [21]) and segmental strain was calculated by applying an automated tracking algorithm. Peak systolic GCS and GLS were calculated as the average strain of all segments at end-systole in the long- and short-axis views, respectively. Scans were only excluded from the analysis if no view could be analyzed due to insufficient image quality (e.g. GCS could not be determined due to insufficient image quality of the 2-,3- and 4-chamber images). Figure 3 shows exemplary images of the same volunteer at the three sites, as displayed on the scanner and after post-processing.

### Intra- and inter-observer reproducibility analysis

Measurements were repeated in a subset of three random volunteers per site (9 volunteers = 36 scans) by the first observer two months after the first analysis and by a second observer who received the same training by software representatives beforehand, blinded to all previous strain measurements. Before repeating the analysis, both observers came to a consensus of excluding volunteers, if both observers considered no view to have the sufficient image quality to determine either GCS or GLS reliably.

### Statistical analysis

The distribution of all values was assessed for normality using the Shapiro-Wilks test. Normally distributed data is expressed as mean ± standard deviation, non-normally distributed data using median and interquartile range (IQR). Inter-vendor agreement between the three sites was determined using Bland-Altman analysis. Test-retest reproducibility between averaged scans before (average strain of scan 1 and 2) and after the fifteen-minute break (average strain of scan 3 and 4) and between single scans was determined using intraclass correlation (ICC) and coefficients of variation (CoV). Wilcoxon test (for non-normally distributed strain parameters) and paired students t-test (for normally distributed strain parameters) were calculated to determine if differences in strain values between the sites and before and after the break were significant. Intra- and inter-observer reproducibility were analyzed using ICC and CoV. The following levels of agreement were used: excellent for ICC > 0.74, good for ICC 0.6–0.74, fair for ICC 0.4–0.59 and poor for ICC < 0.4 [2, 22]. All values are expressed using p-values and confidence intervals. A p-value of ≤ 0.05 was considered significant in two-tailed tests. Statistical analyses were conducted using SPSS (Version 25.0, IBM Corp., Armonk, NY, USA).

## Results

fSENC-image acquisitions of the gel-phantoms were repeated several times. Mean strain and standard deviation were − 28.1% (± 0.3) for the system used at site I, − 23.7 (± 0.9) for the system used at site II and − 26.8 (± 1.4) for the system used at site III. Table 1 portrays the baseline characteristics of the volunteers, vital signs and median (IQR) strain values. One complete fSENC-examination including all images was acquired in a median (IQR) scan time of two (1–4) min at all sites. Median image analysis time ranged from 10 to 14 min for one whole examination. A total of four scans were performed for each volunteer (twice before and twice after the break). At site I, one scan had to be excluded from GLS-analysis owing to motion artifacts during acquisition of the short-axis images*.* At site II, one volunteer could not be scanned due to unexpected technical difficulties. Further four scans were excluded from GLS- and nine from GCS-analysis because of artifacts that would not allow reliable contouring of the heart. At site III, no scan was excluded. A total of 51 scans (85.0%) were left for GLS-and 47 scans (78.3%) for GCS-analysis.

**Table 1:** Baseline characteristics of the volunteers (n = 15), median (IQR) scan time and median strain values (IQR) at the different sites

Volunteer characteristics	Site I	Site II	Site III
Female, n (%)	8 (53%)	8 (53%)	8 (53%)
Age (years)	25 (± 5)	25 (± 5)	25 (± 5)
Height (cm)	174 (± 9)	173 (± 8)	174 (± 9)
Weight (kg)	66 (± 11)	66 (± 11)	66 (± 11)
Smoking, n (%)	2 (13%)	3 (20%)	3 (20%)
Blood pressure before exam (mmHg)	123 (± 18)/68(± 11)	129 (± 18)/74 (± 9)	123 (± 16)/64 (± 9)
Blood pressure after exam (mmHg)	112 (± 17)/61(± 7)	127 (± 15)/70(± 7)	120 (± 16)/62(± 10)
Heart rate before exam (bpm)	74(± 12)	77 (± 15)	67 (± 12)
Heart rate after exam (bpm)	69(± 7)	75 (± 11)	76 (± 9)
Scan time (min.)	2 (1–2)	3 (2–6)	3 (1–4)
LV-GLS (%) (n = 51)	− 19.2 (− 20.5 to − 18.0)	− 17.8 (− 20.0 to − 16.4)	− 17.9 (− 20.0 to − 16.0)
LV-GCS (%) (n = 47)	− 19.7 (− 21.1 to − 18.3)	− 18.9 (− 20.0 to − 17.1)	− 18.2 (− 19.2 to − 16.8)

### Inter-vendor agreement

**Fig. 4:**
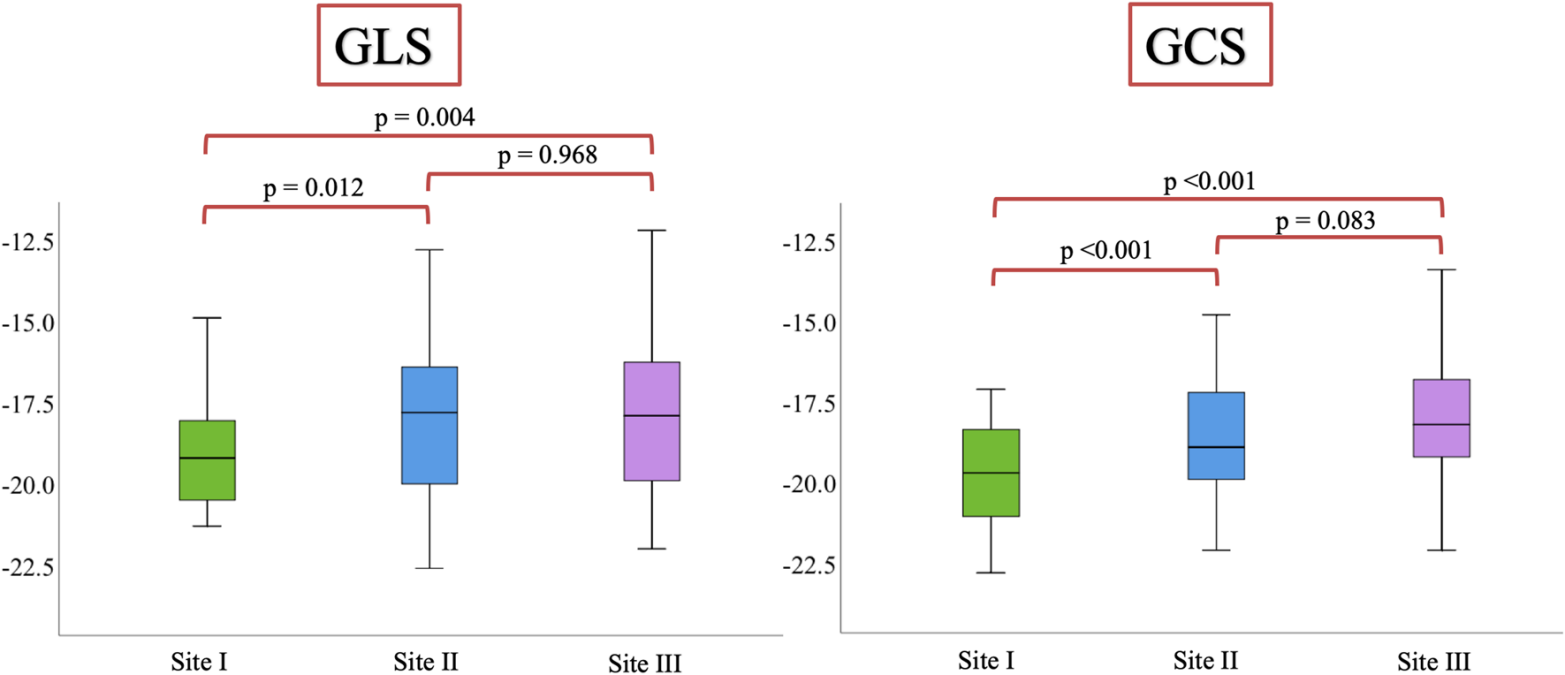
Box and whisker-plots to illustrate the range of strain values with regard to the different sites and the significance level of the differences

**Fig. 5:**
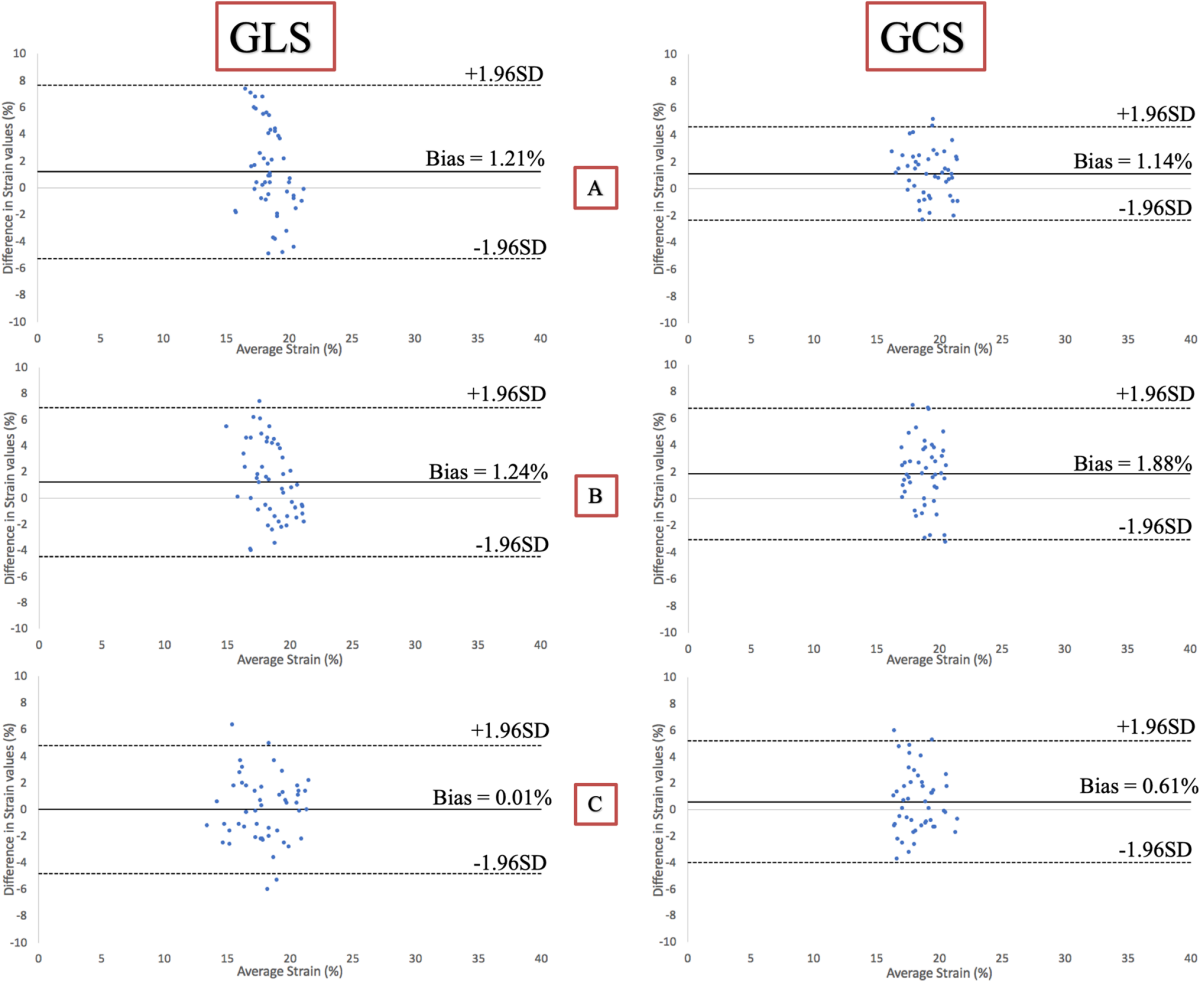
Bland-Altman analysis comparing GLS and GCS between the different sites. Legend: **a** Site I vs. II, **b** Site I vs. III, **c** Site II vs. III

Figure 4 shows box and whisker-plots to illustrate the range of strain values with regard to the different sites and the significance level of the differences, as calculated from the Bland-Altman analysis. The range of GLS-measurements was wider than of GCS-measurements. Differences in strain values were significant when comparing site I against either site II or III. Table 2 and Fig. 5 display the results of the Bland-Altman analysis. Inter-vendor agreement was good between all sites, shown by small biases (0.01–1.88% strain), but the limits of agreement (LOA) reflected a possible inconsistency regarding individual patients. Biases and limits of agreement were significant when comparing site I against either site II or III.

**Table 2:** Results of the Bland-Altman analysis illustrating inter-vendor agreement

	Bias (%)	LOA (%)	p
LV-GLS (n = 51)			
Site I vs. II	1.21	− 5.25 to 7.68	0.012
Site I vs. III	1.24	− 4.47 to 6.92	0.004
Site II vs. III	0.01	− 4.78 to 4.81	0.968
LV-GCS (n = 47)			
Site I vs. II	1.14	− 2.34 to 4.64	< 0.001
Site I vs. III	1.88	− 3.02 to 6.79	< 0.001
Site II vs. III	0.61	− 3.99 to 5.20	0.083

### Test-retest reproducibility

Table 3 displays the median (IQR) strain values of the averaged scans before and after the break and the corresponding p-value, as well as the ICC (95% CI) and CoV (± sd). As shown by the good- to excellent ICC- and CoV-values, test-retest reproducibility of averaged scans before and after the break was very high for all sites. The highest test-retest reproducibility was observed for LV-GLS at site II (ICC = 0.97) and the lowest for LV-GLS at site I (ICC = 0.63). At site I, test-retest reproducibility was higher for GCS-measures, whereas at site II and III, it was higher for GLS-measures. Nevertheless, differences in median strain between scans before and after the break were mostly insignificant (except for LV-GLS for site I). Table 4 shows the scan-rescan reproducibility between single scans. Overall, scan-rescan reproducibility was good, independent of scanner site (ICC = 0.97–0.70). The highest scan-rescan repeatability could be observed for site II between scans 3 and 4 and 1 and 3, the lowest regarding site I between scans 1 and 3.

**Table 3:** Median (IQR) strain before and after the 15-min break at every site and results of the ICC (95% CI) and CoV (± sd) to display test-retest reproducibility

		Median (IQR) strain	p	ICC (95%CI)	p	CoV (± sd)
Site I	LV-GLS	− 20.1 (− 20.9 to − 18.3)	0.020	0.63 (0.21 to 0.86)	0.002	0.06 (± 0.05)
		− 19.4 (− 20.6 to − 17.8)				
	LV-GCS	− 19.0 (− 21.1 to − 18.3)	0.950	0.82 (0.53 to 0.93)	< 0.001	0.05 (± 0.03)
		− 19.4 (− 21.0 to − 18.3)				
Site II	LV-GLS	− 19.9 (− 21.3 to − 16.8)	0.347	0.97 (0.90 to 0.99)	< 0.001	0.03 (± 0.02)
		− 20.1 (− 21.2 to − 17.0)				
	LV-GCS	− 17.4 (− 19.0 to − 16.4)	0.307	0.80 (0.47 to 0.94)	< 0.001	0.04 (± 0.04)
		− 17.3 (− 18.6 to − 16.7)				
Site III	LV-GLS	− 18.8 (− 20.2 to − 15.1)	0.977	0.82 (0.54 to 0.94)	< 0.001	0.09 (± 0.07)
		− 18.4 (− 19.8 to − 16.2)				
	LV-GCS	− 17.2 (− 18.6 to − 16.3)	0.056	0.69 (0.29 to 0.88)	0.001	0.07 (± 0.05)
		− 18.5 (− 19.5 to − 16.5)

**Table 4:** Scan-rescan reproducibility, represented using ICC (95% CI) and CoV

		ICC (95%CI)	p	CoV (± sd)
Site I	GLS			
	Scan 1 vs. 2	0.94 (0.81 to 0.98)	< 0.001	0.03 (0.02)
	Scan 2 vs. 3	0.75 (0.16 to 0.92)	0.002	0.07 (0.04)
	Scan 3 vs. 4	0.97 (0.91 to 0.99)	< 0.001	0.02 (0.02)
	Scan 1 vs. 3	0.70 (0.15 to 0.90)	0.013	0.06 (0.06)
	Scan 1 vs. 4	0.74 (0.27 to 0.91)	0.007	0.06 (0.06)
	Scan 2 vs. 4	0.79 (0.33 to 0.93)	0.002	0.07 (0.05)
	GCS			
	Scan 1 vs. 2	0.89 (0.68 to 0.96)	< 0.001	0.05 (0.06)
	Scan 2 vs. 3	0.83 (0.47 to 0.94)	0.002	0.07 (0.06)
	Scan 3 vs. 4	0.86 (0.56 to 0.95)	0.001	0.05 (0.04)
	Scan 1 vs. 3	0.79 (0.37 to 0.93)	0.004	0.06 (0.04)
	Scan 1 vs. 4	0.86 (0.58 to 0.95)	< 0.001	0.05 (0.04)
	Scan 2 vs. 4	0.88 (0.62 to 0.96)	< 0.001	0.06 (0.05)
Site II	GLS			
	Scan 1 vs. 2	0.88 (0.61 to 0.96)	< 0.001	0.07 (0.07)
	Scan 2 vs. 3	0.94 (0.80 to 0.98)	< 0.001	0.04 (0.05)
	Scan 3 vs. 4	0.97 (0.91 to 0.99)	< 0.001	0.03 (0.03)
	Scan 1 vs. 3	0.97 (0.89 to 0.99)	< 0.001	0.04 (0.03)
	Scan 1 vs. 4	0.94 (0.81 to 0.98)	< 0.001	0.06 (0.04)
	Scan 2 vs. 4	0.95 (0.77 to 0.99)	< 0.001	0.05 (0.04)
	GCS			
	Scan 1 vs. 2	0.94 (0.79 to 0.98)	< 0.001	0.04 (0.04)
	Scan 2 vs. 3	0.89 (0.61 to 0.97)	0.001	0.05 (0.03)
	Scan 3 vs. 4	0.85 (0.52 to 0.96)	0.001	0.05 (0.04)
	Scan 1 vs. 3	0.79 (0.20 to 0.95)	0.013	0.06 (0.04)
	Scan 1 vs. 4	0.85 (0.46 to 0.96)	0.002	0.04 (0.06)
	Scan 2 vs. 4	0.85 (0.50 to 1.00)	0.002	0.05 (0.05)
Site III	GLS			
	Scan 1 vs. 2	0.92 (0.77 to 0.97)	< 0.001	0.08 (0.09)
	Scan 2 vs. 3	0.84 (0.51 to 0.95)	0.001	0.10 (0.11)
	Scan 3 vs. 4	0.96 (0.90 to 0.99)	< 0.001	0.06 (0.05)
	Scan 1 vs. 3	0.89 (0.67 to 0.96)	< 0.001	0.09 (0.09)
	Scan 1 vs. 4	0.89 (0.68 to 0.97)	< 0.001	0.10 (0.08)
	Scan 2 vs. 4	0.85 (0.55 to 0.95)	0.001	0.10 (0.08)
	GCS			
	Scan 1 vs. 2	0.90 (0.70 to 0.97)	< 0.001	0.06 (0.03)
	Scan 2 vs. 3	0.71 (0.18 to 0.90)	0.012	0.08 (0.06)
	Scan 3 vs. 4	0.85 (0.56 to 0.95)	0.001	0.06 (0.05)
	Scan 1 vs. 3	0.71 (0.12 to 0.90)	0.005	0.08 (0.06)
	Scan 1 vs. 4	0.79 (0.38 to 0.93)	< 0.001	0.08 (0.05)
	Scan 2 vs. 4	0.83 (0.50 to 0.94)	0.001	0.07 (0.05)

### Intra- and inter-observer reproducibility

Both observers independently excluded one volunteer out of nine from strain analysis, resulting in 32 scans. Intra- and inter-observer reproducibility were very high overall (Table 5), but even higher for GLS than for GCS.

**Table 5:** Intra- and inter-observer reproducibility, reflected by ICC (95% CI) and CoV (± sd)

	ICC (95%CI)	p	CoV (± sd)
Intra-observer reproducibility			
LV-GLS	0.99 (0.98 to 1.00)	< 0.001	0.02 ± 0.02
LV-GCS	0.77 (0.47 to 0.90)	< 0.001	0.05 ± 0.04
Inter-observer reproducibility			
LV-GLS	0.96 (0.92 to 0.98)	< 0.001	0.03 ± 0.04
LV-GCS	0.82 (0.58 to 0.92)	< 0.001	0.04 ± 0.03

## Discussion

It has been shown that strain, determined using either echocardiography or CMR, is a valuable parameter to determine the impact of coronary artery disease on heart function [12], to detect LV dysfunction, especially in patients with heart failure when EF is still preserved [1, 4, 12, 23] and to reveal diffuse damage to the myocardium due to systemic diseases, such as cardiac amyloidosis [24, 25], sarcoidosis [26] or cardiotoxic effects of chemotherapy [5]. Despite these many possible indications, the use of strain in clinical routine is still challenging due to the impact of intra-, inter-observer- [7] and inter-vendor reproducibility of the different post-processing platforms [8, 9, 27] on strain results, which could also explain the lack of inter-technique agreement between echocardiography and CMR [28]. Therefore, before conducting studies to validate strain techniques in large patient cohorts, it is important to (1) identify the possible factors influencing strain results and to (2) minimize the impact of these factors. To address this issue, we compared GLS and GCS in healthy volunteers, who were all scanned using fSENC at three different sites with MRI scanners from major vendors.

Our results show:

1. good inter-vendor agreement of strain measurements using fSENC between all three vendors overall, reflected by small biases but substantial limits of agreement
2. very good test-retest reproducibility of fSENC when scanning volunteers again after a fifteen-minute break, regardless of vendor, and
3. good to excellent intra- and inter-observer reproducibility of fSENC strain measurements.

To our knowledge, no previous data on inter-vendor agreement of a CMR-technique to determine strain exists. Nevertheless, the influence of different ultrasound systems on 2D- and 3D-STE has been reported previously [7, 29–31]. As in our study, differences in STE-strain measurements between the different vendors were significant [7, 29, 30]. However, the bias between different ultrasound systems was similar or higher (0.1–3.7 [7], 1.1–7.0 [30], 1–1.55% [31]) than the bias between magnetic resonance scanners determined in our study group of fifteen volunteers (0.01–1.88%), with limits of agreement of a similar magnitude. The bias in our cohort of healthy volunteers was significant between site I and II or III. Moreover, the limits of agreement indicate that in some individuals the difference in strain values could be considerably higher than the bias. We believe that this study demonstrates the importance of further exploring inter-vendor agreement in larger cohorts to validate these results and to determine the agreement related to different scanners in patients. Our results indicate that it might possibly be helpful to implement scanner-related normal values and that one should be aware of this possible bias and limits of agreement when comparing strain results acquired at different scanners. This should also play a role when designing classifications based on strain, which determine diagnostic procedures and therapeutic decisions for patients.

An important factor that could influence inter-vendor agreement is the difference in technical characteristics of the pulse sequence at the different scanners. A spiral readout was used at sites I and II, whereas an EPI was used at site III, which may have different properties in terms of geometric distortion and susceptibility to off-resonant spins. Furthermore, the pulse sequence varied with regard to most scanning parameters for each scanner. In order to determine the influence of the pulse sequence alone on strain measurements, phantoms were scanned at sites with the three different scanning systems before scanning the volunteers. Mean strain values of the phantoms were higher using the scanning systems at site II and III than using the system at site I, similar to the pattern of median GLS and GCS of the volunteers. This suggests that the pulse sequence itself could contribute to differences in strain values. Other possible variables with impact on inter-vendor agreement are the planning and training of different technicians, the experience and training of the observers and changes in the physiology of the volunteers. In order to minimize the effect of differences in knowledge and training of the technicians and observers in our study, all received training on image planning/analysis and completed written tests. Furthermore, a standardized imaging protocol was used at all three sites, but technicians were allowed to adjust the scanning parameters. Additionally, if two technicians performed the scanning, different levels of experience and planning styles resulted in different image planning at the same scanner. Due to the above listed reasons, the scans were of variable quality, which may have affected strain measurements. To monitor and reduce volunteer-related bias, volunteers were asked questions regarding their health, medications and smoking behavior before every scan and height, weight, blood pressure and heart frequency were monitored. Volunteers with new onset of disease or new intake of medication would have been excluded, but the impact of changes in factors such as weight and smoking behavior on strain measurements were not ruled out. In addition, it was not possible to keep the time difference between the scans at the three sites consistent, so we could not eliminate changes in myocardial function associated with timing of the scans. However, previous literature studying temporal variability of T_1_- and T_2_ mapping in volunteers after approximately 90 days [32] and 4D flow in 10 volunteers with a difference of one year between scans [33] reported no significant differences or significant agreement of results, indicating that myocardial function in healthy volunteers should be stable over a certain time period up to 1 year. Furthermore, the volunteers were also scanned at different time-points during the day, allowing for short-term differences in loading conditions to possibly affect strain results. Nevertheless, we only observed minor changes in volunteer characteristics, vital parameters and CMR-parameters, so we assume that myocardial function was stable in our group of volunteers during the course of the study.

The good to excellent test-retest reproducibility of averaged scans before and after the break and between single scans observed in our group of volunteers, regardless of MRI scanner used, matches the excellent test-retest reproducibility Giusca et al. reported in fSENC scans of eleven healthy subjects and seven patients with heart failure repeated 63 days apart [2]. These results also suggest that effects of short-term differences in myocardial function relating to loading conditions, stroke volume and heart frequency are minimal in volunteers. Furthermore, the very good to excellent intra- and inter-observer reproducibility we reported agrees with previous studies investigating fSENC [2, 15] as well.

When comparing CMR techniques to measure strain, obstacles preventing broad clinical use are centered around the long acquisition and post-processing time, especially concerning myocardial tagging [15, 34]. Due to the fast image acquisition without the need for breath-holds, fSENC could be a potential alternative to tagging. Strain measurements using fSENC have already been shown to be valuable to detect hypertrophic cardiomyopathy when EF is preserved [35], right-ventricular dysfunction due to pulmonary hypertension [36] and diastolic dysfunction in patients with type II diabetes mellitus [37]. Furthermore, fSENC reliably identifies myocardial regions affected by coronary artery disease and infarction [38] and reliably estimates LV-volumes and EF in patients with coronary artery disease, as shown by a recent study from our group [39].

### Clinical implications

Our results suggest that an average bias of 0.01% to 1.88% strain (< 1.24% for GLS and < 1.88% for GCS) should be taken into account when comparing fSENC results of healthy individuals acquired using different scanners. This implies that a strain difference of below 2% on average may represent normal variability in the measurement and not necessarily a decrease or increase in myocardial function, if scanning is performed using different scanners. The limits of agreement indicate that strain results from different scanners should not be used totally interchangeably. Larger studies are needed for further validation in order to facilitate the planning and comparison of multi-center studies, which are needed for standardization of strain measurements and to determine inter-vendor agreement in patients. Furthermore, technical differences between different scanners and imaging sequences should be assessed.

### Limitations

Our study group is composed of a relatively small sample size of healthy young volunteers, in order to eliminate the influence of pathologies on strain measurements. Hence, it is important to conduct further studies to assess inter-vendor agreement in a larger study cohort and in patients. Furthermore, in-vitro scanning was performed using different phantoms, at different sites than where the volunteers were scanned and with different number of repeats per site. Unfortunately, multiple scans at site II had to be excluded from further strain analysis due to technical complications that similarly occur in the clinical routine, such as a defect optical fiber cable (preventing one volunteer from being scanned) and a malfunctioning body coil, resulting in artifacts during four GLS and five GCS scans. Additionally, we only focused on fSENC in this study and did not include conventional tagging, the gold standard for strain measurements, since fSENC had previously been validated against tagging [15]. Similarly, we did not evaluate other techniques for measuring strain. Nevertheless, it would be interesting to examine the impact of different MRI scanners on other CMR techniques used to determine strain, including tagging.

## Conclusion

We found good inter-vendor agreement of strain measurements acquired with the fSENC technique at 3 T using MRI scanners from three major vendors with small biases, but considerable limits of agreement and a significant difference in strain results. Test-retest reproducibility between repeated scans was very high, regardless of the scanner chosen. Moreover, reproducibility of strain measurements was good to excellent, independent of the employed MR-platform. fSENC can be considered a reliable technique and suitable for strain measurements at different centers and, with further development, has the potential to improve diagnostics and therapy in heart failure patients. Our results might help to interpret strain assessed by fSENC at different sites using MRI scanners from different vendors.

## Abbreviations

CMR: Cardiovascular magnetic resonance
EF: Ejection fraction
EPI: Echo planar imaging
fSENC: Fast strain-encoded magnetic resonance imaging
GCS: Global circumferential strain
GLS: Global longitudinal strain
IQR: Interquartile range
LV: Left ventricular
LOA: Limits of agreement
MRI: Magnetic resonance imaging
SAX: Short-axis
SENC: Strain-encoded magnetic resonance imaging
STE: Speckle tracking echocardiography
2-Ch: 2-Chamber
3-Ch: 3-Chamber
4-Ch: 4-Chamber
